# Necrosis

**Published:** 1896-08

**Authors:** N. W. Hiatt

**Affiliations:** Marion, Ind.


### Necrosis.

BY DR. N. W. HIATT, MARION, IND.

Read before the Indiana State Dental Society, Indianapolis, Ind., June 30, 1896.

Necrosis of bone, and caries of bone, while seemingly the same, differ slightly in action. Caries of bone might be designated as the molecular disintegration of bone, or the death of an osseous structure in particles, which, as a destructive process, resembles the ulcerative action peculiar to the soft tissue, and is a slow process; while necrosis is a more rapid process of bonedeath which destroys considerable portions of osseous material at a time, and is analagous to sloughing of the soft tissues.

In taking up the study of necrosis, we are first impressed by the fact that necrosis does not, in itself, constitute a disease, but is the result of previous inflammatory disease of the bone.

According to Stanley, in his treatise on "Diseases of the Bones" and the order of frequency in which necrosis occurs in different bones of the body, we find the lower jaw more susceptible than the upper, the upper jaw being one of the last to be affected. An examination as to the difference in structure of the two jaws shows the lower to be very compact, and the upper very cancellous. The frequency of necrosis in lower jaw as compared with the upper may be attributed to the compact nature of the bone, and the inability of the inflammatory products to find their way to the surface; while just the opposite may be said of the upper jaw, which is very cancellous, and as a result the inflammatory products find their way to the surface without much resistance from the thin layers of bone covering the roots of the teeth.

The injurious effect of tension is thus lessened, and in a great many cases entirely prevented. Heath tells us that "the blood supply has an important part to play as to the difference of the suspectibility of the two jaws to be attacked by necrosis."

The upper jaw is supplied by numerous branches of the internal maxillary artery, which inosculates freely from side to side, and in this way nature is enabled to carry away the inflammatory product and resist the poisonous effects.

There seems to be a difference of opinion as to the blood-supply of the lower jaw. By some writers it is claimed that the arteries supplying the lower jaw do not anastomose, a condition which, if it does not exist—and I am inclined to doubt it—would make quite a difference as compared with the upper jaw, where they do anastomose. I have here some specimens of bone removed, and as they are passed around, I will explain the case to you as best I can.

Case No. 1: Was of the right superior maxillary bone, and was accompanied with abscess of antrum. When patient first called on me he complained of pain in first molar tooth. Face was slightly swollen; a large cavity on mesial approximate surface; cavity was opened and pulp found dead. Caustic pyrozone was used to clean roots and patient dismissed. The next day I was asked to call at the house, as the patient's face was so swollen that he could not come out. On examining the mouth I found what I thought to be an ordinary abscess resulting from the molar tooth I had treated, yet there was no tenderness in the tooth, which was still very firm. I opened abscess, and washed in the usual way. On the next day I called again and found not only the abscess-pockets refilled with pus, but the mucous membrane of the palate, as far as the mesial line, was swollen down even with the grinding surfaces of the teeth. Both places opened and cleansed and the patient told to call at the office on the following morning, which he did, but with no improvement. Teeth were getting slightly loose, but not tender to pressure. No discharge of pus into roots of molar, so they were filled and abscess cleansed again, and patient told to come again the next morning. At this visit there was quite a little pain in the first bicuspid tooth, the second bicuspid being gone. Tooth was so loose that we removed it immediately and found quite a discharge from the antrum. On examination of the tooth extracted, I found under a cement filling a dressing of iodoform. Nerve had been destroyed and this dressing put in, but patient had never been back to have it finished. This he told me after I discovered the dressing. The opening into the antrum was enlarged and caustic pyrozone injected into it. There was no pain as a result of it, but pyrozone came out of his nose, and, seemingly, all over his mouth. This treatment was kept up three days longer, but the teeth were getting looser all the time, and had I taken a pen and ink I am sure I couldn't have drawn a line showing more plainly the line of demarkation than, shown between the two superior maxillary bones and the mucous membrane covering them. The right central was so loose that extracting it with the fingers would have been an easy matter, while the left one was immovable. Swelling had almost disappeared, but pus was coming from around all the teeth on the right side. I removed them and found the bone, in places, had separated, and was only hanging in the gum tissue. The bone was removed to the floor of the antrum; part of that, however, we were able to save. The discharge of pus stopped immediately, and the patient was entirely well within ten days or two weeks. There was, of course, some deformity which might be overcome to a certain extent with a partial denture.

Case No. 2: Patient had a left inferior first molar treated, nerves taken out and filled, something over a year ago. Was at Atlanta, Georgia, where work was done. Tooth ulcerated and abscess formed, and opened shortly afterward, and this condition had existed ever since. The fact that there was constant drainage, there was no pain, but tooth was loose. The patient came to me because the left side of his face, and left side of lower lip was continually numb, and, being a cornet-player, bothered him quite a little. Upon examination I found the inferior dental nerve entirely gone. The section of bone taken out extended from first the bicuspid to the third molar, and about a quarter of an inch below the inferior dental canal. The specimen I show you is the only large piece removed, the remainder was scraped and burred away. The molar tooth that had been treated, I am sorry to say, was kept by the patient. When I decided to operate on his jaw, he promised to stay under my care a week, but when the engagement of his company was over two days later, he was getting along so nicely he decided to go away with them, and promised to let me hear from him. He wrote me from over in Illinois that he was all right as far as he knew; that his jaw had healed nicely, and had no pain at all.

Within the last six months I have had two cases of necrosis so similar to the ones just mentioned that it will not be necessary to mention them.

Since I have been a member of this Association I don't remember of hearing anything said on this subject, and if the discussion will bring out anything to enlighten me, that I may be better able to take care of it when necessary, I shall feel that I am surely repaid for my trouble.

